# Myocardial tagging in the polar coordinate system; early clinical experience

**DOI:** 10.1186/1532-429X-16-S1-P387

**Published:** 2014-01-16

**Authors:** Sarah N Khan, Abbas N Moghaddam, Razieh Kaveh, Adam Plotnik, Evan Lehrman, Ali Nsair, J Paul Finn

**Affiliations:** 1 Radiology, UCLA, Los Angeles, California, USA; 2 Biomedical Engineering, Tehran Polytechnic, Tehran, Iran, Islamic Republic of

## Background

Quantitative MR myocardial strain analysis is typically performed using rectilinear or Cartesian grid tagging, and regional contractility is visually assessed by the deformation of the grid1. However, it is difficult to visually isolate circumferential and radial components of displacement and strain from parallel straight lines on short axis images. This study evaluates the potential of a polar coordinate tagging system2 for quantification of circumferential myocardial displacement in a variety of clinical conditions.

## Methods

33 subjects including 12 patients with congenital heart disease (CHD), 11 with cardiomyopathy (CM) and 10 healthy volunteers (HV) underwent cardiac SSFP cine and myocardial tagging in the mid-ventricular short axis plane with grid tags (line separation 5-7 mm), circular tags (5 mm separation) and radial tags (11 lines per semi-circle; 18 degree separation). Visual assessment of circumferential and radial displacement was performed by two observers for each of the six mid short axis segments. Scores were assigned for: overall tag quality (4 point scale), strain (5 point scale; normal +2, hypokinesia 1, akinesia 0, mild dyskinesia -1, severe dyskinesia -2), confidence in the findings (3 point scale) and ease of interpretation (4 point scale). Quantitative analysis of global and segmental circumferential strain was performed using a semi-automated tool previously described3.

## Results

**Figure 1:**
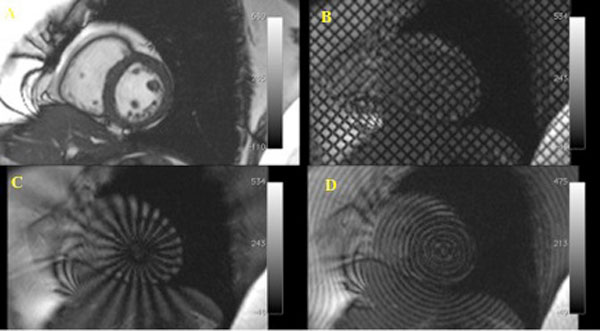
**Images from a healthy volunteer**. (A) SSFP Cine image in the mid ventricular slice (B) Cartesian grid tagging pattern (C) Radial tagging pattern (D) Circular tagging pattern.

Healthy volunteers (Figure 1) had the highest global circumferential strain (-0.0836 ± 0.0111), followed by CHD (-0.0781 ± 0.0157) and CM (-0.0620 ± 0.0192). Regional circumferential strain was highest in the inferolateral segment in all three groups (HV -0.105 ± 0.0342, CM -0.118 ± 0.0306, CHD -0.139 ± 0.0381). Regional circumferential strain was lowest in the anteroseptal segment in the CHD group (0.0596 ± 0.0316), anteroseptal and inferoseptal segments in the CM group (0.0429 ± 0.0477 and -0.0600 ± 0.0482), anteroseptal and anterior segments in the HV group (-0.0571 ± 0.0279 and -0.0608 ± 0.0659). Healthy volunteers had the highest scores for tag quality, strain, confidence and ease of interpretation, followed by CHD and CM patients. Polar tagging was easier to interpret (average score 3.6) than grid tagging (average score 2.9).

## Conclusions

Polar tagging has advantages for the visual and quantitative assessment of myocardial strain. Compared to rectilinear tagging, radial tags are easier to interpret for isolation of circumferential and radial components of myocardial motion. Overall tag quality, strain, confidence in assessment and ease of interpretation of the image were highest in healthy volunteers, second highest CHD patients and lowest in CM patients.

## Funding

n/a.

